# Milk and dairy products – a scoping review for Nordic Nutrition Recommendations 2023

**DOI:** 10.29219/fnr.v68.10486

**Published:** 2024-12-30

**Authors:** Kirsten Bjørklund Holven, Emily Sonestedt

**Affiliations:** 1 Department of Nutrition, Institute of Basic Medical Sciences, University of Oslo, Oslo, Norway; 2 Nutritional Epidemiology, Department of Clinical Sciences Malmö, Lund University, Malmö, Sweden

**Keywords:** milk, dairy, cheese, yogurt, dietary recommendations

## Abstract

Milk and dairy products are major sources of protein, calcium, and other micronutrients. Milk and dairy products contribute with approximately half of the total intake of saturated fat in the Nordic and Baltic diets. Saturated fat is an important determinant of plasma total and low density lipoprotein (LDL)-cholesterol concentrations, and a causal relationship between high LDL-cholesterol and atherosclerotic cardiovascular disease has consistently been documented. The aim of this scoping review is to describe the evidence for the role of milk and dairy products for health-related outcomes as a basis for setting and updating food-based dietary guidelines. Two qualified systematic reviews were included (World Cancer Research Fund and a systematic review for the US Dietary Guidelines Advisory Committee 2020). In addition, systematic reviews published between January 2011 and January 2022 were considered, screened (555 records) and evaluated (159 records) for this review. The systematic reviews suggest that milk or dairy consumption is not associated with increased risk of cardiovascular disease and dyslipidaemia. Current evidence suggests an inverse association with some cardiometabolic risk factors, such as total and LDL-cholesterol, especially regarding fermented dairy products (i.e. yogurt and cheese). There was evidence of an association between intake of dairy products and reduced risk of colorectal cancer. Some studies reported an inverse association between intake of dairy and type 2 diabetes or markers of impaired glucose homeostasis, especially for low-fat dairy, yoghurt, and cheese. Most studies suggest that intake of milk or dairy is not associated with increased risk of cardiovascular risk and some suggestions of inverse association, especially with low-fat products and fermented dairy products, were found with respect to cardiovascular disease, type 2 diabetes, and colorectal cancer. Milk or dairy products are important dietary sources of calcium and iodine, and are fully compatible with a healthy dietary pattern.

## Popular scientific summary

- Milk and dairy products are good sources of protein and essential amino acids, vitamin A, riboflavin, vitamin B12, calcium, and iodine.
- Milk and dairy products are also major sources of saturated fatty acids in Nordic and Baltic diets.
- Intake of dairy products is associated with lower risk of colorectal cancer.
- Milk and dairy products in general are not associated with risk of cardiovascular disease, but may modestly reduce blood pressure.
- Low-fat and fermented dairy products, such as yogurt and cheese, may be associated with lower serum cholesterol and risk of type 2 diabetes.
- Health effects of milk and dairy products can differ based on both the nutrient composition and the structure of the products.

Dairy products are a heterogenous group of products. Milk from ruminants is both a food and a raw material for different dairy products such as cheese, cream, butter, and fermented milk (e.g. yogurt and sour milk). The fat content in milk varies from 0.1 g to around 4 g per 100 g, the protein content (whey and casein) is about 3.0–3.5 g per 100 g, and the carbohydrate content (disaccharide lactose consisting of glucose and galactose molecules) is about 4–5 g per 100 g. Whole milk and low-fat milk have the same proportions of fatty acids. Two-thirds of the fatty acids in milk consists of saturated fatty acids (SFA), mainly myristic (C14:0), palmitic (C16:0), and stearic (C18:0) acid. Milk also contains short-chain fatty acids, and small amounts of the odd-chain fatty acids C15:0 and C17:0 which are almost exclusively found in milk products. The main unsaturated fatty acid is oleic acid (C18:1). In addition, small amounts of trans-fatty acids are produced by bacteria in one of the cow’s stomachs (rumen), which makes milk the major source of trans-fats in countries where the industry-produced trans-fats have been reduced or eliminated. In fermented milk (i.e. yogurt and sour milk) selected bacteria are added and part of the lactose has been converted to lactic acid and other acids. Cheese contains essentially the same nutrients as milk except lactose. Cream is concentrated milk fat and therefore contains lower amounts of water-soluble vitamins, protein, and lactose.

Milk and dairy products are good sources of protein and essential amino acids, vitamin A, riboflavin, vitamin B_12_, calcium, and iodine. Milk, yogurt, and cheese are the foods with the highest amounts of calcium. Whole-fat and low-fat milk contain about the same amounts of calcium (120 mg per 100 g), and cheese contains 750–940 mg calcium per 100 g. Around 60% of the calcium intake in the Nordic and Baltic diet is from milk products (1). Although milk products are generally good sources of micronutrients, they usually contain very little iron. In the Nordic countries, it is either mandatory or voluntarily with vitamin D fortification in milk (2). However, the products that are fortified and the amounts differ between countries. Compared to milk, the plant-based milks (e.g. from soy, oat, almond, rice, and pea) have a low content of many micronutrients and proteins. Currently, several plant-based milks fortified with calcium, vitamin B_12_, and vitamin D are available.

Dairy products contribute approximately 50% of the saturated fat intake in the habitual diet in Nordic countries. A causal relationship between LDL-cholesterol and atherosclerotic cardiovascular disease (CVD) has consistently been documented (3, 4). Replacing SFA with polyunsaturated fat (PUFA) reduces risk of coronary heart disease (5–7) and effectively reduces plasma cholesterol levels (7, 8). Importantly, dairy products differ in their nutritional content as well as their physical structure. For example, cheese is high in saturated fat, but its composition is more like milk and yogurt than to that of butter. Because of differences in composition and structure, the health effects of various dairy foods may differ. For example, myristic and palmitic acids increase LDL-cholesterol concentrations more than lauric acid, while stearic acid has a neutral effect (9). The health effects are also likely more than the sum of the nutrients in dairy foods. In a systematic review for the Nordic Nutrition Recommendations (NNR) 2012, there was limited-suggestive evidence for total dairy consumption to be associated with decreased risk of type 2 diabetes, and suggestive evidence for total dairy consumption to be associated with increased risk of prostate cancer and decreased risk of colorectal cancer (10). However, since the health effects of different types of dairy products may differ, the aim of this scoping review is to describe the totality of evidence for the role of milk and dairy products for health-related outcomes as a basis for setting and updating food-based dietary guidelines in NNR 2023 (Box 1).

Box 1 Background papers for Nordic Nutrition Recommendations 2023

- This paper is one of many scoping reviews commissioned as part of the Nordic Nutrition Recommendations 2023 (NNR2023) project (11)
- The papers are included in the extended NNR2023 report but, for transparency, these scoping reviews are also published in Food & Nutrition Research
- The scoping reviews have been peer reviewed by independent experts in the research field according to the standard procedures of the journal
- The scoping reviews have also been subjected to public consultations (see report to be published by the NNR2023 project)
- The NNR2023 committee has served as the editorial board
- While these papers are a main fundament, the NNR2023 committee has the sole responsibility for setting dietary reference values in the NNR2023 project

## Methods

This scoping review follows the protocol developed within the NNR2023 project (11). The following qualified systematic reviews were identified by the NNR2023 Committee (12) and were included as source of evidence: The World Cancer Research Fund (WCRF) report (13) and one systematic review for the 2020 US Dietary Guidelines Advisory Committee (14). In addition, a literature search was performed in the PubMed database using the following search; (milk [MeSH Terms] OR dairy products [MeSH Terms] OR cheese [MeSH Terms]) AND (“2011”[Date – Publication] : “3000”[Date – Publication]) AND humans[Filter] AND (systematic review[Publication Type] OR meta-analysis[Publication Type]). Systematic reviews and meta-analysis published between January 2011 and January 2022 were considered, 555 publications were screened, and 159 abstracts were evaluated. The sources of evidence used in the review follow the eligibility criteria described previously (15). Finally, a separate search in PubMed was conducted in March 2022 on Mendelian randomization studies using the lactase persistence genotype as a proxy for long-term intake of lactose-containing dairy, using the following terms: (Mendelian randomization[Title/Abstract]) AND ((LCT[Title/Abstract]) OR (lactase[Title/Abstract]) OR (milk[Title/Abstract])). We included publications from meta-analyses or consortia. Only articles covering outcomes predefined for NNR2023 were selected. Out of 36 publications, 8 articles were included as sources of evidence (16–23) out of which 4 articles were also identified in the previous search.

## Diet intake in Nordic and Baltic countries

Intake of milk and dairy products from the most recent national dietary surveys (year 2010–2020) is shown in Table 1. Intake of milk and dairy products ranges from 124 g/day among women in Lithuania to 480 g/day among men in Finland. Intake of cheese ranges from 15 g/day among women in Estonia to 46 g/day among men in Norway.

**Table 1:** Consumption of milk and dairy products in the Nordic and Baltic countries.

Country, year (age group)	Gender	Milk and dairy products, g/day	Cheese, g/day
Denmark, 2011 (18–75 y)	Men (*n* = 1,464)	337 (274)	41 (35)
	Women (*n* = 1,552)	273 (197)	41 (31)
Finland, 2017 (18–74 y)	Men (*n* = 780)	480 (449–512)	
	Women (*n* = 875)	394 (373–415)	
Iceland, 2010 (18–80 y)	Men (*n* = 632)	353 (266)	
	Women (*n* = 680)	251 (182)	
Norway, 2010 (18–70 y)	Men (*n* = 862)	384 (354)	46 (44)
	Women (*n* = 925)	249 (237)	42 (38)
Sweden, 2010 (18–80 y)	Men (*n* = 792)	267 (231)	25 (28)
	Women (*n* = 1,005)	227 (171)	25 (28)
Estonia, 2014 (18–74 y)	Men (*n* = 907)	294 (220)	20 (22)
	Women (*n* = 1,806)	252 (223)	15 (23)
Latvia, 2020 (19–64 y)	Men (*n* = 470)	224 (222)	
	Women (*n* = 541)	185 (156)	
Lithuania, 2019 (19–75 y)	Men (n = 1,348)	140 (163)	
	Women (*n* = 1562)	124 (131)	

Data are provided as mean and SD or 95% Confidence Interval (Finland). Data is retrieved from respective country’s most recent national dietary survey in adults.

## Health outcomes

In the current review, we focused on the association between dairy intake and cardiovascular risk, type 2 diabetes, cancer, osteoporosis, hypertension, overweight/obesity and all-cause mortality. The main findings are described in more detail below.

### Dairy and cardiovascular disease or risk factors

We identified 25 new systematic reviews published since 2010 regarding intake of dairy and CVD or cardiovascular risk factors, such as plasma total and LDL-cholesterol (10, 16, 24–43) and metabolic risk factors. Five of the systematic reviews studied the effect of dairy or specific dairy products on cardiovascular risk factor including total and LDL cholesterol. Two systematic reviews including only randomized studies found either no significant effect of dairy products on neither LDL or high density lipoprotein (HDL) cholesterol (24), or that consumption of dairy can either increase or decrease lipids dependent on the specific type of dairy product (28). One systematic review investigating only the effect of cheese found that cheese reduced total, LDL and HDL cholesterol compared to butter (25). The systematic reviews overall conclude that milk or dairy consumption is not associated with increased risk of CVD. Most of the meta-analyses investigating effects on CVD found neutral or favorable association (27, 36, 37) with intake of dairy products or specific dairy products; yogurt (44), cheese (25, 31), and fermented dairy (40). Few studies have directly compared the effect of low- versus high-fat dairy products; however, one meta-analysis investigated the consumption of full-fat or low-fat dairy products and found no association between either type of dairy and all-cause mortality and CVD incidence (45), whereas one systematic review showed that intake of high-fat milk compared to low-fat milk was associated with increased CVD risk (31).

### Dairy and cancer

We identified 31 new systematic reviews (17, 44–74). WCRF reported that there was strong evidence that intake of dairy products reduces the risk of colorectal cancer (75). There was limited evidence suggesting that dairy products decrease the risk of premenopausal breast cancer and limited evidence for a positive association between high intake of dairy products and risk of prostate cancer (75).

### Dairy and risk of type 2 diabetes, overweight, and obesity

We identified eight systematic reviews regarding the intake of dairy and diabetes (76–83). An inverse association between intake of dairy and type 2 diabetes or risk factors for type 2 diabetes (e.g. Homeostatic Model Assessment for Insulin Resistance; HOMA-IR) was suggested for most studies, specifically for low-fat dairy, yogurt, and cheese. Regarding intake of dairy and overweight/obesity, we identified eight systematic reviews (84–91). However, the evidence is insufficient and inconsistent regarding the association between intake of dairy foods and risk of obesity or body weight gain. The 2020 US Dietary Guidelines Advisory Committee concluded that there is limited evidence suggesting that high milk consumption is not associated with adiposity in neither children nor adults (92).

### Dairy and bone health

We identified eight systematic reviews and/or meta-analyses published regarding the intake of dairy and bone health or markers of bone health (18, 93–99). Evidence for beneficial effects for adults was inconsistent and limited, but the overall evidence does not support a link between dairy consumption and fractures. There is some evidence from intervention studies that milk intake in childhood and adolescent age results in faster linear growth and a positive effect on bone health.

### Dairy and other health markers

We identified six new systematic reviews and/or meta-analyses published regarding the intake of dairy and hypertension or blood pressure (19, 100–104). No evidence for a detrimental effect related to intake of dairy products was seen. A modest, beneficial effect on blood pressure was reported in most studies. We identified 10 new systematic reviews and/or meta-analyses study published regarding the intake of dairy and all-cause mortality (35, 37, 105–112). Most of the studies found no association between total dairy intake and all-cause mortality.

### Mendelian randomization studies using genetic variation as a proxy for long-term milk intake

The Mendelian randomization analyses provide support for a causal association between lactose-containing dairy (i.e. milk) intake and higher BMI, lower LDL and HDL cholesterol (16), higher waist circumference, lean body mass (20), and higher insulin (23). Mendelian randomization analyses suggest a reduced risk of colorectal cancer with high milk intake, but there is no or limited evidence that milk consumption affects the risk of bladder, breast, and prostate cancer (17). There is no support that genetically predicted adult milk intake is associated with systolic blood pressure (19), hip fractures (18), bone mineral density, ischemic heart disease, or type 2 diabetes (21, 23).

## Mechanisms

### Cardiovascular disease

Although milk and dairy products are major sources of SFA, which increases plasma total, LDL- and HDL cholesterol levels compared with carbohydrates, dairy consumption has not been shown consistently to increase total and LDL cholesterol. However, replacing full-fat dairy products with low-fat products is associated with a dietary pattern that is more beneficial for cardiovascular health (11). Dairy products consist of very heterogenous food groups with different composition. While some dairy types, for example, milk, yogurt, cheese, and fermented dairy foods, are associated with a neutral or favorably lipid profile, butter and full-fat milk are associated with an unfavorable lipid profile. In addition to the long-chain SFA, they also contain medium- and odd-chain SFA, trans-fatty acids, unsaturated fatty acids, branched-chained amino acids, other nutrients (e.g. calcium, iodine, vitamin K), and bioactive components such as bioactive peptides, which all are potentially mediating different effects. Moreover, dairy foods also have different other characteristics with some dairy products being fermented, being differently processed, and the dairy matrix is different. All these differences can modulate their bioavailability and subsequently their metabolic effect. Calcium and bioactive peptides have been suggested to mediate beneficial effects, for example, on blood pressure and blood cholesterol, while some dairy products also have been suggested to influence markers of inflammation (113, 114). A recent review concluded that milk or dairy products did not show any proinflammatory effect in healthy subjects or individuals with metabolic abnormalities (114). In addition, other beneficial mechanisms have been suggested, such as reduced lipid absorption (by different food matrix) and microbial fermentation producing short chain fatty acids such as butyrate (45). Therefore, because dairy foods being such a heterogenous group of products containing numerous different nutrients influenced by their specific matrix, it is not easy to draw a firm conclusion on their health effects. More studies are needed to determine the effect of specific dairy products on CVD risk.

#### Cancer

The mechanisms involved in the possible inverse association between dairy products and colorectal cancer are at present unclear. The high calcium content of dairy products has been attributed for the protective effect (13). Fatty acids and bile acids in the colon may play a role in the initial phase of the carcinogenesis, and calcium could protect by sequestration of secondary bile acids. Conjugated linoleic acid present in dairy foods has also been suggested to be protective, for example, by inhibiting cell proliferation, decreasing inflammatory mediators, and stimulating the immune system (48).

### Diabetes, overweight, and obesity

Several mechanisms have been put forward regarding the potential beneficial effect of dairy products on type 2 diabetes risk. Milk proteins, such as whey, may have insulinotropic activity, and calcium is essential for insulin secretion by β-cells. Dairy foods contain short- and medium-chain fatty acids that has been suggested to possess a range of biological activities including induction of hormones influencing insulin sensitivity and impact the microbiota, regulate energy metabolism, improve β-cell function, and reduce inflammation (115). Consumption of dairy may facilitate maintaining body weight and fat loss because of the content of calcium, protein (casein and whey), and other bioactive compounds in dairy foods that may favorably affect energy balance. Calcium interacts with fatty acids in the intestine and may thereby reduce absorption of fat. Full-fat dairy products, such as butter, (sour) cream, and full-fat cheese, are energy-dense foods, and high intake of these may lead to energy surplus. Several dairy products contain probiotic bacteria that interact with the normal bacteria flora. Some fermented dairy products seem to have a more beneficial effect on circulating inflammatory markers than non-fermented dairy products (116).

### Bone health

Osteoporosis is defined as reduced bone mineral density and increased bone fragility, and is a major public health problem. In the Nordic countries, milk and dairy products are important sources of nutrients associated with bone health, first and foremost calcium and vitamin D. Calcium is an important component of the skeleton, and vitamin D is involved in absorption of calcium from the intestine and the regulation of calcium metabolism, leading to increased bone mineralization (117).

## Food-based dietary guidelines

### Summary

Dairy products are a heterogenous group of products including, for example, milk, cheese, and yogurt. New evidence suggests that effects on health outcomes mediated by dairy products differ and extend beyond their individual nutritional composition. Because dairy products contain animal fat, with a large proportion being saturated fat, high consumption of high-fat dairy products can lead to high intake of saturated fat. This may contribute to an increased risk of CVD. However, milk or dairy consumption has not been associated with increased risk of CVD. Current evidence suggests inverse associations with cardiometabolic risk, especially regarding fermented dairy products (i.e. yogurt and cheese). In addition, an inverse association with intake of dairy and type 2 diabetes has been reported, specifically for low-fat dairy, yogurt, and cheese. However, replacing full-fat dairy products with low-fat products is associated with a dietary pattern more beneficial for cardiovascular health. There is evidence for an association between intake of dairy products and reduced risk of colorectal cancer, and some evidence for a protective effect of dairy food on risk of obesity.

Dairy products (especially milk, yogurt, and cheese) are important sources of calcium, protein, iodine, and other important nutrients. To satisfy the need for calcium, 6 deciliters of milk alone or 2–5 dL a day of milk, depending on what other foods are included in the diet, is sufficient. The amount of fat varies substantially between dairy products. To avoid consuming too much saturated fat, low-fat milk, and cheese should preferably be chosen. Dairy products that are not a major source of essential nutrients nor associated with health effects, that is, cream and butter, should be limited.

Rather than assuming that two dairy foods with similar nutritional content have the same health effects, the health effects of specific dairy foods should be considered. Thus, the physiological effects of a dairy food cannot necessarily be predicted solely based on its nutritional content or fat composition. The evidence suggests that the health effects of different dairy products vary, even for dairy foods with high fat content (i.e. butter and cheese), and the food matrix and content of bioactive compounds need to be considered to clarify the potential beneficial effect of dairy products.

### Data gaps for future research

Several new systematic reviews and meta-analyses have been published since NNR 2012, and a few of these have looked at specific dairy products (e.g. cheese and yogurt). However, there are still too few studies to draw firm conclusions. Current evidence suggests no association between milk and dairy intake and CVD, and the consequence of shifting from low- to more full-fat dairy could be explored further.

Different dairy products may possess different effects depending on fermentation degree, matrix nutrient composition, and bacteria strain used; therefore, more studies on the effect of the different dairy products are needed. Moreover, little focus has been on systematically comparing the effect of low- versus full-fat dairy because most studies compare different dairy products to another or another food source containing saturated fat. In addition, there is a need to compare plant-based dairy alternatives to cow’s milk on health outcomes.

Finally, there is also a need for more studies using objective biomarkers of dairy consumption, including circulating biomarkers or genetic markers.
